# Variation in the Measurement of Anti-Müllerian Hormone – What Are the Laboratory Issues?

**DOI:** 10.3389/fendo.2021.719029

**Published:** 2021-09-03

**Authors:** Rivak Punchoo, Sachin Bhoora

**Affiliations:** ^1^Tshwane Academic Division, National Health Laboratory Service, Pretoria, South Africa; ^2^Department of Chemical Pathology, Faculty of Health Sciences, University of Pretoria, Pretoria, South Africa

**Keywords:** Anti-Müllerian Hormone, AMH, Müellerian inhibiting substance, MIS, standardisation, analytical interference

## Abstract

Anti-Müllerian Hormone (AMH) is a 140 kDa homodimeric glycoprotein consisting of two identical subunits linked by disulphide bonds and is synthesised by the testes and ovaries. Its clinical applications are prediction of ovarian response and gonadotropin dose selection upon *in vitro* fertilization. In males, AMH is used to investigate sexual developmental disorders and gonadal function. AMH is commonly assayed by enzyme-linked immunosorbent assay or automated immunoassay formats that show variation between methods. This review applies fundamental chemical pathology concepts to explain the observed analytical variation of AMH measurement. We examine the lack of standardisation between AMH assays, the impact of antibody design on variable measurements, consider the analytical detection of AMH isoforms, review analytical interference in AMH measurement, and briefly assess systematic bias between AMH assays. The improved attempt at standardising AMH measurement by the recent approval of a WHO Reference Reagent offers promise for harmonising immunoassay results and establishing consensus medical cut-off points for AMH in disease. Standardisation, however, will need to redress the issue of poor commutability of standard reference material and further assign a standard reference procedure to quantify AMH standard reference material. The improvement of the analytical phase of AMH testing will support harmonised method development and patient care.

## Introduction

Müllerian Inhibiting Substance (MIS), also known as Anti-Müllerian Hormone (AMH), is a dimeric glycoprotein that is a member of the transforming growth factor-beta superfamily (1). AMH is synthesised by Sertoli and granulosa cells of the testes and ovaries, respectively (2). AMH regulates antral follicles in the human ovary before final selection and is the gatekeeper of follicular oestrogen production and selection of the dominant follicle that will undergo ovulation (3, 4). In the testes, AMH production triggers the regression of Müellerian ducts in the male foetus and is also involved in testicular development and function (5).

The clinical applications of AMH are ovarian reserve testing (6), prediction of ovarian response after controlled ovarian stimulation, prediction of the menopause onset, monitoring of ovarian effects of medication and surgical procedures, and evaluating the risk of a variety of ovarian disorders, for example, polycystic ovary disease and primary ovarian failure (7). It is also used in the investigation of male sexual developmental disorders and gonadal function.

Clinical applications require analysis along an analytical range that can accommodate accurate and reliable measurement at high and low levels of the reference interval to enable accurate diagnosis and disease monitoring. Currently, multiple immunoassays are available to quantify AMH and variability in measuring AMH is commonly reported (6, 8).

An electronic literature search was performed for the present review using PubMed and Google Scholar databases. The search terms used were: “AMH”, “MIS”, “Measurement”, “Method comparison”, “Method evaluation”, “Analytical interference”. The inclusion criteria were studies related to humans and studies which investigated AMH measurement. The exclusion criteria were articles for which full text was not available, not written in English, were grey literature, or original articles published before the last 20 years. Core textbooks in chemical pathology prescribed by the College of Medicine of South Africa were also consulted. Studies retrieved from initial searches were supplemented with additional references that were identified by manual search among the cited references.

This review will examine issues that account for variation in serum AMH measurement, emphasising the analytical phase of the total testing laboratory process. Basic concepts in chemical pathology that underly measurement variation of AMH will also be explained to provide non-laboratory trained readers with essential laboratory knowledge to rationalise the observed variations in AMH measurement. The critical issues we will explore are standardisation of AMH assays, antibody design and detection employed in AMH measurement, analytical interference, limits of detection for manual and automated AMH testing, and systematic error between AMH assays.

## Quantification of AMH by Commercial Assays

The measurement of AMH immunoassays has evolved over the last two decades. Assay formulations have utilized various calibrators and antibody pairs for quantification of AMH. Consequently, the bias, imprecision, and limit of detection (LoD) demonstrate variability. Newer assays utilize automated formats with improved analytical sensitivity. **Table 1** summarizes key analytical characteristics of current assays commonly utilized for AMH measurement.

**Table 1:** Analytical characteristics of immunoassays currently used to measure AMH (9–13).

Assay parameters		Gen II ELISA	Ultra-sensitive AMH/MIS ELISA	MenoCheck^®^ picoAMH ELISA	Access AMH	Elecsys^®^ AMH Immunoassay
	Manufacturer	Beckman Coulter Diagnostics (Texas, USA)	Ansh Laboratories (Texas, USA)	Ansh Laboratories (Texas, USA)	Beckman Coulter Diagnostics (Texas, USA)	Roche Diagnostics International Ltd. (Indiana, USA)
Assay format	Test format	ELISA (2 site manual immunoassay)	Sandwich type ELISA	Sandwich type immunoassay	Automated immunoassay	Automated immunoassay
	Primary antibody	Anti-AMH IgG immobilised to microtiter plate well	AMH antibody coated to microtiter plate	Biotinylated antibody coated microtiter plate	Mouse monoclonal anti-AMH antibody alkaline phosphatase conjugate	Antibody-antigen ‘sandwich’ complex with two mammalian monoclonal antibodies conjugated to biotin and ruthenium
	Secondary antibody and/or conjugation system	Antibody-biotin conjugate and streptavidin -enzyme conjugate	Biotinylated anti-AMH secondary antibody	Streptavidin-HRP conjugate	Chemiluminescent substrate	Ruthenium/tripropylamine conjugate
	Detection system	TMB chromogen substrate	SHRP conjugate TMB substrate	TMB substrate	Chemiluminescent detection	Chemiluminescent detection
Calibrators	Calibrator traceability		Manufacturer’s working calibrators	Internal calibrator is traceable to rhAMH		
	Calibrator levels	7 levels	6 levels	6 levels	Multipoint calibration	
	Calibrator concentration range	0 - 22.5 ng/ml	Not disclosed	0 - 1.100 pg/ml		
	Matrix			Protein-based matrix		
Assay sensitivity	Limit of detection (LoD)		0.023 ng/ml	1.3 pg/ml	≤ 0.02 ng/ml	0.010 ng/ml
	Limit of quantification (LoQ) with < 20% CV		0.06 ng/ml	3.2 pg/ml	≤ 0.08 ng/ml	0.030 ng/ml
Assay imprecision (based on maximum CV at low and high AMH levels)	Intra-assay CV (%) [AMH]	5.4% (4.42 ng/ml) 3.4% (16.45 ng/ml)	6.03 (0.72 ng/ml)	5.5% (14.6 pg/ml) 3.9% (935 pg/ml)		1 – 2.6% (0.046 - 20.8 ng/ml)
	Inter-assay CV (%); [AMH]	5.6% (4.42 ng/ml) 4% (16.45 ng/ml)		6.7% (15.5 pg/ml) 5.4% (942.8 pg/ml)		2.5 – 3.9% (0.046 - 20.8 ng/ml)
	Total imprecision CV (%); [AMH]	7.7% (4.42 ng/ml) 5.3% (16.45 ng/ml)			< 10% (≥ 0.16 ng/ml)	< 5%
Measuring range		0.16- 22.5 ng/ml 1:10 dilution available		6.0 – 1.150 pg/ml	0.08 – 24 ng/mL	0.01 – 23 ng/ml
Extended measuring range with dilution step				23.000 pg/ml	240 ng/ml	

CV, coefficient of variation; ELISA, enzyme-linked immunosorbent assay; HRP, horseradish peroxidase; rhAMH, recombinant human AMH; SHRP, streptavidin horseradishperoxidase; TMB, tetramethylbenzidine.

## Lack of Calibration of AMH Assays Against an International Standard Using a Reference Method Contributes to Inter-Assay Bias

Immunoassays, including enzyme-linked immunosorbent assay (ELISA) format, are calibrated by defined analyte material (calibrators) to allow quantification of analyte concentration in unknown patient samples and quality control materials (14). A calibration curve can be constructed for both manual and automated immunoassays using manufacturer-assigned values to calibrators. Multiple calibrators (two or more) are used to establish a dynamic range of measurement from low to high concentration levels, which span relevant medical decision points. Therefore, the standard curve defines the assay’s measuring range and links the concentration and signal output of the calibrators to the analytical signal from unknown samples or controls. Patients’ samples and control material are quantified by extrapolating the assay signal to the concentration using a calibration curve (or its straight-line equation). A concentration value is then assigned to the unknown sample. The use of a reference procedure and reference material defines the metrological principle of standardisation. It links the patient’s test result to the pure material, ensuring a line of traceability (14–16).

AMH measurement suffers from variable assay standardisation between commercial immunoassays (8, 17). Manufacturers of AMH assays have used assay-defined proprietary calibrators derived from various sources with variably assigned values. This causes variation of standard curves between AMH assays and has contributed to the observed variation of AMH measurement by immunoassays. Reference material either from the International Standards by the WHO or Standard Reference Materials (SRM) certified by the National Institute of Standards and Technology (NIST) exist only for a limited number of measurands. This is because standardisation is expensive, demanding human resources and onerous to establish as it consists of multi-stage processes. The designation of appropriate higher-order methods to quantify pure standard material enables manufacturers to assign values to their calibrators. Thus patients results are directly traceable to the high order reference material (15).

The establishment of an international standard to calibrate AMH immunoassays will enable harmonisation of AMH testing across multiple commercial assays, standardise proficiency testing schemes, allow clinical laboratories to calibrate and control immunoassays, and support continued research and development in the measurement of AMH. The WHO Expert Committee approved the development of an international standard for AMH immunoassays in 2014. Recombinant AMH trial preparation SS-581 showed stability in lyophilised form and retained biological activity in the Müllerian duct regression assay (17).

Following on this study, a stable, lyophilised preparation of recombinant, CHO-derived AMH, encoded 16/190, was assessed by a collaborative international panel by multiple AMH immunoassays (18). Most assays detected the AMH preparation; however, unsurprisingly, considerable variation between assays was noted, ascribed to the variable method calibrators used by the individual assays. The various manufacturer calibrators are derived from native, recombinant, and non-human sources. Some assays exhibited low variability of AMH content in a comparator sample to the 16/190 preparation content estimate. The WHO assigned a mass to 16/190 based on a mean consensus amongst agreeable immunoassays.

The commutability of the AMH 16/190 material, however, was unsatisfactory. Commutability is a property of reference material that describes the equivalence of the mathematical relationships between the results of different measurement procedures for reference material and representative samples from healthy and diseased individuals (19, 20). Thus, for routine diagnostic laboratories, commutability requires validation across all assays and methods that use the reference material. This ensures that patients’ results by routine measurement procedures, for example, AMH measured by automated or manual immunoassays, have equivalent values regardless of the AMH immunoassay used for the measurement. Commutability is, therefore, an essential requirement when a reference material is to be used as a common calibrator for clinical laboratory assays or in proficiency testing schemes or by commercial manufacturers as part of their internal traceability procedures to assign value to their product calibrators. Ultimately, commutability allows evaluation of the agreement amongst various measuring procedures of patient results. As commutability was unsatisfactory for 16/190, the WHO approved 16/190 as a WHO Reference Reagent, rather than as “full” WHO International Standard (18). The reasons for poor commutability include the lack of inclusion of all AMH isoforms in the 16/190 preparation, variable epitope recognition of 16/190 by immunoassay antibodies between methods in the study, and poor alignment of the non-human protein matrix of 16/190 with serum.

Furthermore, until a physicochemical reference method becomes available, the AMH content of 16/190 will be defined as 489 ng/ampoule, which is the mean of the estimates from a subset of valid AMH immunoassays (18). This assignment is an essential step in moving to a fully standardised reference material and reference measurement procedure for AMH measurement. In the interim, the international reagent preparation will assist commercial AMH manufacturers in re-evaluating the assignment of manufacturer calibrator values and improving harmonised comparisons between AMH immunoassays.

## AMH Isoforms Complicate Measurement – Are the Relevant Biologically Active AMH Forms Being Measured?

AMH is synthesised as a precursor hormone with a signal sequence followed by the pre-hormone segment (pro-AMH). AMH contains two cleavage sites at amino acid 229 and 451 and, when cleaved, forms a family of AMH isoforms. Proteolysis of pro-AMH results in the formation of a 58 kDa N-terminal “pro-region” domain (AMH_N_), and a biologically active 12.5kDa “mature” C-terminal domain (AMH_C_). Each 70 kDa homodimer dimerises and is linked non-covalently by disulphide bonds to form the 140 kDa AMH_N,C_ hormone, which is glycosylated and secreted (21, 22). Commercial AMH assays target various parts of the AMH hormone. Capture and detection antibodies of ELISAs are assay-specific and, for example, target mature region and pro-region of AMH (16).

Biological activity is attributed to AMH_C_ and AMH_N,C_ isoforms. AMH-C-terminal homodimer is less active than the non-covalent complex. Restoration of the activity is achieved by associating the C-terminal homodimer with the N-terminal homodimer implying that the N-terminal domain functionally amplifies the C-terminal domain. This suggests that the AMH isoforms possess differential biological activity. The AMH non-covalent complex represents the most potent bioactive form of AMH, which can bind optimally to AMH receptor II (AMHRII) (23–26).

Detection of inactive, partially active, and fully active measurands by non-specific antibodies increase total measurand concentration and produces false positive results. The ratios of circulating AMH isoforms are incompletely elucidated. Biologically inactive pro-AMH and bioactive dimerised AMH_N,C_ are both detected in blood circulation by current AMH immunoassay technology (6, 27). The quantification of total serum AMH thus represents a summation of biologically active and inactive isoforms. Therefore, the total AMH laboratory result may spuriously suggest an increased bioactive fraction of AMH that may potentially misclassify patients’ clinical states.

Antibodies are proprietary showing differential sensitivity and specificity to detect AMH isoforms in disease. The capture and detection antibodies in ELISA target unique epitopes on AMH. Therefore, the manufacturer’s specification of which isoforms are being measured will accurately estimate active AMH.

In addition, the proportion of isoforms may also vary between diseases, which will affect the quantification of total AMH. However, studies analysing the ratio of different isoforms in gonadal pathology and in healthy state are required to inform advocating selective assays to measure specific circulating isoforms (28, 29). Post-translational modification of AMH in disease needs clarification. Using the Ansh lab manual ELISA, proAMH in normo-ovulatory women was shown to constitute 3% of promature AMH (proAMH + AMH_N,C_) and similar studies exploring isoform detection is required (28).

Furthermore, various assay matrices, for example, with varied pH and ionic concentration, can alter the 3-dimensional structure of AMH isoforms and their detection. The assay matrix may also then influence AMH measurement. Therefore, isoforms in an assay-specific serum matrix for calibrators is crucial to reduce AMH measurement variation (6).

## Variability in Antibody Specificity Causes Variation in AMH Detection

The evolution of antibody design for the detection of AMH from the 1990s to the present time shows changes in two areas, viz clonal selection of antibodies and variable AMH antigenic material used to raise antibodies. Furthermore, the changing demand of AMH clinical utility from a marker of testicular function to the assessment of ovarian function, ovarian reserve and disease has demanded improved limit of detection for AMH in females. This has necessitated the re-design of capture and detection antibodies for AMH immunoassays.

The various antibodies used have impacted test sensitivity. For instance, early immunoassays used polyclonal and monoclonal antibodies against the pro-AMH region of recombinant human AMH (rhAMH) and/or bovine AMH, achieving sensitivities of 0.5 ng/ml – 6.25 ng/ml (30, 31). Later ELISA tests detected total AMH by raising polyclonal and/or monoclonal antibodies to rhAMH. The capture and detection antibodies were raised against the pro-AMH and the mature regions of AMH, and therefore able to detect total AMH. The IOT assay sensitivity for the monoclonal antibody pair was 0.1 ng/ml (32) in comparison to the assay utilising a combination of monoclonal and polyclonal antibodies, and the limit of detection was 2 ng/ml (33).

In a cross-sectional study comparing AMH levels among three commercially available AMH immunoassays (AMH Gen II, Beckman Coulter; Ultrasensitive AMH, AnshLab; and picoAMH, AnshLab), significantly higher proportions of detectable AMH levels were observed with the picoAMH assay (97%) and Ultrasensitive AMH assay (92%) compared to Gen II assay (84%) (34). The AnshLab utilises similar antibody pairs for both its ELISA tests compared to Gen II assay, and antibody selection may contribute to the observed differences.

The continued development of antibody design for ELISAs for glycoprotein hormones will need to consider variation in specificity, cross-reactivities, epitope locations (35) and clinical application. Achieving agreement about relevant biological AMH isoforms will improve the specificity of AMH detection and the inter-assay agreement. Knowledge of antibody specificity will enable targeting equimolar detection of AMH isoforms; however, sharing proprietary antibody pairings is challenging but would undoubtedly standardise medical decision cut-off points.

## Analytical Interference in AMH Immunoassays

Immunoassays suffer analytical interference from a broad range of sources, including heterophile antibodies, human anti-animal antibodies, serum proteins (e.g. rheumatoid factor, binding proteins), drugs and drug metabolites, and abnormal serum indices (e.g. haemolysis, lipaemia and hyperproteinaemia). The laboratory usually flags significant interference; however, low-level interference may be undetected and adversely affect test interpretation (36–38). As AMH is run only on immunoassay format, it is susceptible to varied analytical interference.

The use of biotin-streptavidin-based immunoassays has demonstrated positive and negative analytical interference in competitive and non-competitive sandwich immunoassays, respectively (39, 40). Many AMH assays use biotin-streptavidin based measurement and are at risk for this type of interference. Platforms that use alternative methods of antigen capture do not show this analytical interference (40, 41). Manufacturers’ instructions on maximum tolerable concentrations of *in vivo* biotin levels, guidelines on biotin supplementation and time-to-sample interval, re-formulation of biochemical assays, and studies investigating biotin interference have collectively improved biotin immunoassay interference.

Complement levels can interfere with AMH measurement. The unmodified AMH Gen II produced erroneously low AMH concentrations, especially in fresh patient samples. The interference resulted from the binding of C1q to the capture antibody IgG2a and subsequently activating the complement cascade and c3b deposition, causing steric hindrance preventing AMH-binding to the capture antibody (42). This interaction was favoured by the assay matrix, which allowed complement activation and by the use of a capture antibody that strongly activated complement. Introducing a pre-dilution step to the revised version of AMH Gen II improved complement interference, although it may cause positive bias (43). Interestingly, the automated Beckman Access and Roche Elecsys AMH assays are not affected by complement interference despite using the same antibody pairs as the Beckman AMH Gen II assay (40–42).

## Sensitivity, Limit of Detection and Imprecision Show Variability Between Manual and Automated AMH Assays and Can Impact Clinical Applications

The improvement in assay imprecision and LoD has evolved with newer automated AMH assays. These automated immunoassays also utilise chemiluminescent detection, which increases the assay sensitivity and permits low-level AMH detection. For example, Elecys^®^ AMH Immunoassay (Roche Diagnostics International Ltd, Indiana, USA) and the Access AMH assay (Beckman-Coulter Diagnostics (USA) demonstrate respective LoD of 0.01 ng/ml and 0.02 ng/ml (11, 13).

Using proficiency testing material quantified by ten laboratories by the AMH GenII ELISA over a 15-month scheme, the within-laboratory reproducibility was good; however, the between laboratory variability showed a wide range of average values compared to the consensus mean (-24.0% to +22.7%) (44) emphasising inter-laboratory imprecision of manual ELISA testing. Manufacturer data also identifies a broader imprecision of intra-assay and inter-assay CVs for the AMH GenII ELISA (≤5.4% and ≤5.6%) compared to automated assays: Access AMH (≤1.7% and ≤2.8%) and Elecsys AMH Immunoassay (≤2.6% and ≤3.9%) (11, 13). The Ansh Lab’s picoAMH ELISA has CVs above >5%; however, their Ultra-Sensitive ELISA has CVs <5% (10, 12). It is not surprising that manual ELISA assays are generally less precise as the multi-step human handling requires pipetting small volumes, performing multiple wash steps and carefully timing incubations.

The superior analytical performance of automated assays is especially valuable at fixed cut-off medical decision points to avoid misclassification. For example, patients who receive ovarian stimulation have binary clinical response cut-off points of ≤0.75ng/ml and ≥3.50 ng/ml that identify low and high responses to stimulation (45). The increased detection limits and sensitivity of automated assays allow the determination of AMH at low concentrations. This is illustrated by comparison laboratory evaluation data between the AMH Gen II ELISA assay and the Elecys^®^ AMH assay in orthotopic transplantation of ovarian tissue after gonadotoxic treatment (46). The enhanced analytical performance of automated assays can also benefit tumour marker applications, such as detecting recurrence of granulosa cell ovarian tumours (47–49).

## Comparison of Systematic Error Between AMH Assays

The total analytical error, which accounts for variability between AMH assays, is derived from a summation of random error and systematic error. Random error is unpredictable and can be derived from various sources during the performance of the test in a laboratory, for example, incorrect aspiration of sample volume by automated or manual AMH assays. Random error contributes to the imprecision of an assay expressed mathematically by the coefficient of variation (CV). High CV values indicate wider imprecision and are less desired for an assay. In contrast, systematic error provides a measure of accuracy (bias) and is further subdivided into contributions from a constant error component and a proportional error component. Constant error is independent of the measurand concentration, and proportional error varies with the concentration of the analyte being measured. Statistical regression methods between two assays quantify slope (proportional error) and y-intercept (constant error). The correlation coefficient (R^2^) indicates the closeness of the sample data points to the regression line and provides a measure of random error. A perfect comparison between two assays is a slope = 1, y-intercept = 0 and a R^2^ = 1 (15, 50).

Assay-specific calibrators can reveal systematic error between methods in a method comparison study. The bias of an assay expresses the sample’s value relative to a true value and provides a measure of accuracy between the comparator and reference method. A bias of 0%, for example, indicates that there is a complete agreement of sample measurement between two different assays. **Table 2** summarizes method comparison studies for AMH measurement. In general, these studies demonstrate variability in measurement between AMH assays. Furthermore, the importance of developing high order reference procedures and an international reference material is foregrounded to enable medical laboratories to establish a chain of analytical traceability from the patient’s result back to the primary reference material.

**Table 2:** Method comparison evaluations between commercial AMH assays.

Assay comparison	Regression equation	Analytical and clinical commentary	Reference
**1. Gen II *vs* Elecsys^®^**	Elecsys^®^ = (0.81 X Gen II) – 0.046	• Elecsys^®^ AMH values were substantially lower compared to Gen II assay, emphasising a need for an international AMH calibrator. • Good correlation between Elecsys^®^ and Gen II assays were demonstrated. • The automated Elecsys^®^ demonstrates improved analytical sensitivity, for example, detection of AMH at 0.5 pmol/l. • Automated immunoassay platforms provide rapid testing for routine clinical practice.	(52)
	Elecsys^®^ = (0.68 X Gen II) + 0.769		(51)
	Elecsys^®^ = (0.729 X Gen II) + 0.087		(53)
	Elecsys^®^ = (0.88 X Gen II) - 0.039		(54)
**2. Gen II *vs* Access**	Access = (0.78 X Gen II) + 0.128	• The automated assay correlated with the Gen II (R=0.996), but with improved sensitivity. • Access AMH assay read at a negative bias compared to Gen II. • Large differences in assay calibration can cause patient misinterpretation when different assays are used in the course of patient management. • Development of an international calibrator for AMH is supported.	(53)
	Access = (0.91 X Gen II) – 0.033	• Access AMH assay read lower in comparison to the Gen II assay.	(54)
	Access = (1.001 X Gen II) + 0.341 Dx1800 = (0.9231 X Gen II) + 0.3475	• The Access AMH assay performed similarly on two analysers: Beckman Coulter Gen II and Dx1800. • Correlation studies demonstrate agreement between Gen II and Access assays. • Both Beckman Coulter assays use the same monoclonal antibodies.	(55)
**3. Gen II *vs* Ansh ultrasensitive assays**	Ultrasensitive AMH = (1.4 X Gen II) + 1.7	• Acceptable imprecision CV% < 6.0% and 10.7% for ultrasensitive and picoAMH assays, respectively. • 15 of 22 serum specimens of AMH were detected by the sensitive assays in comparison to Gen II. • Use of recombinant AMH calibrant for Ansh Lab assays decreases the variability between the two Ansh Lab assays and improves standardisation at low AMH levels.	(56)
**4.Gen II *vs* IOT**	Gen II = (1.353 X IOT) + 0.051	• The Gen II assay was developed when the DSL assay and the IOT assay were purchased by Beckman Coulter. The Gen II assay uses antibodies from the DSL assay and AMH calibrators from the IOT assay. • 56 serum samples covering a wide range (1.9 – 142.5 pmol/l) were analysed. AMH read higher on the Gen II assay in comparison to IOT and DSL.	(57)
**5. Gen II *vs* DSL**	Gen II = (1.223 X DSL) -1.270		(57)
**6. Elecsys^®^*vs* Access**	Elecsys^®^ = (0.97 X Access) + 0.003	• The automated assays were calibrated by independent manufacturer calibrators. The assays compared very favourably with each other, even at low AMH concentration. Another small single centre study supports this observation (58). • No bias was noted. Both assays use the same pair of monoclonal antibodies (as Gen II). • Automated assays showed improved correlation with sub-fertile women and perimenopausal women compare to the Gen II assay. • AMH concentration on the automated assays also correlated highly with the number of antral follicles.	(54)
	Access = (-0.05 X Elecsys^®^) + 1.10	• The Access assay gave systematically higher AMH values resulting in misclassification of women (29%) to ovarian stimulation dosing. • While some studies have demonstrated similar AMH values between two assays, others have shown Access measured AMH values to be 5 - 15% higher than those measured on the Elecsys^®^ (53, 59, 60). • These differences highlight the need for AMH reference materials to eliminate these differences.	(61)

Access, (Access AHM, Beckman Coulter Diagnostics, Texas, USA); DSL, Diagnostic Systems Laboratories (Texas, USA); Elecsys^®^, Elecsys^®^ AMH (Roche Diagnostics Laboratories, Indiana, USA); Gen II, AMH Gen II ELISA (Beckman Coulter Diagnostics, Texas, USA); IOT, Immunotech (Marseille, France); n, number of participants; Ultrasensitive AMH, Ultra-sensitive AMH/MIS ELISA (Ansh Laboratories, Texas, USA); vs, versus.

Comparative assay studies on the workhorse AMH GenII ELISA (reference assay) and comparator assays (Access and Elecys^®^) demonstrate variation. Regression analysis showed slopes between 0.68 and 1, and y-intercepts between -0.039 and +0.769, respectively, in the method comparison studies implicating significant systematic error variation between the assays (51, 53–55, 62). Furthermore, based on cut-off medical decision points at concentrations of 1 ng/ml and 5 ng/ml, biases were calculated using regression equations derived from seven assay comparison studies that assessed performance between manual and automated assays. Biases between -25.2% and + 45% were noted between Gen II and the Elecsys^®^ assay, and biases of between -9% to +34% were identified between Gen II and the Access assay (6). Systematic error in AMH assays contributes to assay variability and can be detected by method comparison studies of AMH assays.

## Conclusions

AMH between-method variability can be ascribed to various processes in the analytical phase of testing. The most significant contributor is the lack of international standardised material to ensure uniform calibration. To this end, the assignment of a WHO Reference Reagent is promising in improving comparability between assays. Other factors that influence inter-assay variation include antibody design with variable specificity, incomplete knowledge about which isoform to measure, and method vulnerability to analytical interferences. Future improvement at the analytical phase of testing for AMH will support the safe establishment of comparable medical decision cut-off points between AMH immunoassays.

## Author Contributions

RP conceived the idea for this article. RP and SB conducted the literature search and drafted the manuscript. Both authors contributed to the article and approved the submitted version.

## Conflict of Interest

The authors declare that the research was conducted in the absence of any commercial or financial relationships that could be construed as a potential conflict of interest.

## Publisher’s Note

All claims expressed in this article are solely those of the authors and do not necessarily represent those of their affiliated organizations, or those of the publisher, the editors and the reviewers. Any product that may be evaluated in this article, or claim that may be made by its manufacturer, is not guaranteed or endorsed by the publisher.
